# Differential effects of formoterol on thrombin- and PDGF-induced proliferation of human pulmonary arterial vascular smooth muscle cells

**DOI:** 10.1186/1465-9921-13-109

**Published:** 2012-11-27

**Authors:** Elena A Goncharova, Irene S Khavin, Dmitry A Goncharov, Vera P Krymskaya

**Affiliations:** 1 Pulmonary, Allergy & Critical Care Division, Department of Medicine, University of Pennsylvania Perelman School of Medicine, Philadelphia, PA, USA; 2 Penn Cardiovascular Institute, University of Pennsylvania, Philadelphia, PA, USA; 3 Abramson Cancer Center, University of Pennsylvania, Philadelphia, PA, USA; 4 University of Pennsylvania, Perelman School of Medicine, Translational Research Laboratories, Room 1214, 125 South 31st Street, Philadelphia, 19104, PA

**Keywords:** (R, R) formoterol, ERK1/2, Pulmonary hypertension

## Abstract

**Background:**

Increased pulmonary arterial vascular smooth muscle (PAVSM) cell proliferation is a key pathophysiological component of pulmonary vascular remodeling in pulmonary arterial hypertension (PH). The long-acting β_2_-adrenergic receptor (β_2_AR) agonist formoterol, a racemate comprised of (R,R)- and (S,S)-enantiomers, is commonly used as a vasodilator in chronic obstructive pulmonary disease (COPD). PH, a common complication of COPD, increases patients’ morbidity and reduces survival. Recent studies demonstrate that formoterol has anti-proliferative effects on airway smooth muscle cells and bronchial fibroblasts. The effects of formoterol and its enantiomers on PAVSM cell proliferation are not determined. The goals of this study were to examine effects of racemic formoterol and its enantiomers on PAVSM cell proliferation as it relates to COPD-associated PH.

**Methods:**

Basal, thrombin-, PDGF- and chronic hypoxia-induced proliferation of primary human PAVSM cells was examined by DNA synthesis analysis using BrdU incorporation assay. ERK1/2, mTORC1 and mTORC2 activation were determined by phosphorylation levels of ERK1/2, ribosomal protein S6 and S473-Akt using immunoblot analysis.

**Results:**

We found that (R,R) and racemic formoterol inhibited basal, thrombin- and chronic hypoxia-induced proliferation of human PAVSM cells while (S,S) formoterol had lesser inhibitory effect. The β_2_AR blocker propranolol abrogated the growth inhibitory effect of formoterol. (R,R), but not (S,S) formoterol attenuated basal, thrombin- and chronic hypoxia-induced ERK1/2 phosphorylation, but had little effect on Akt and S6 phosphorylation levels. Formoterol and its enantiomers did not significantly affect PDGF-induced DNA synthesis and PDGF-dependent ERK1/2, S473-Akt and S6 phosphorylation in human PAVSM cells.

**Conclusions:**

Formoterol inhibits basal, thrombin-, and chronic hypoxia-, but not PDGF-induced human PAVSM cell proliferation and ERK1/2, but has little effect on mTORC1 and mTORC2 signaling. Anti-proliferative effects of formoterol depend predominantly on its (R,R) enantiomer and require the binding with β_2_AR. These data suggest that (R,R) formoterol may be considered as potential adjuvant therapy to inhibit PAVSM cell proliferation in COPD-associated PH.

## Background

Pulmonary arterial vascular smooth muscle (PAVSM) cell proliferation is one of the key pathophysiological components of vascular remodeling in pulmonary hypertension (PH) [1,2]. PH is a common complication of chronic obstructive pulmonary disease (COPD), which is strongly associated with decreased quality of life, increased morbidity and reduced survival of COPD patients [3,4]. The major pathological manifestations of PH are vasoconstriction and remodeling of small muscular pulmonary arteries (PA). Prolonged exposure to hypoxia, growth factors and pro-inflammatory cytokines induces PAVSM proliferation and pulmonary vascular remodeling leading to persistent elevation of pulmonary vascular resistance, right ventricular failure and death [2,5,6]. Systemic vasodilators, however, have not been found to be effective therapy for COPD-associated PH [6] and therapeutic options to target pulmonary vascular remodeling are needed.

β_2_ adrenoreceptor (AR), a member of the G-protein coupled receptor family, is the major subtype of βAR in SM cells. Binding with β_2_AR agonists induces β_2_AR coupling with G_s_ proteins, activation of adenylate cyclase and increase of cellular cAMP levels leading to parallel activation of protein kinase A (PKA) and Epac1 that synergize in mediating cAMP-dependent growth inhibition of VSM cells [7-11] suggesting that β_2_AR agonists may be considered as an attractive therapeutic approach to inhibit PAVSM cell proliferation in PH.

Formoterol is a long-acting β_2_AR agonist that is commonly used as a bronchodilator to treat patients with COPD [12,13]. Formoterol is available in two formulations: racemic formoterol that consists of equal amounts of (R,R) and (S,S) enantiomers, and purified (R,R) formoterol. (R,R) formoterol has 1000-times greater affinity to β_2_AR than (S,S) enantiomer and shows improved bronchodilator effects compared to formoterol racemate [8]. Recent data demonstrate that, in addition to its function as a bronchodilator, racemic formoterol also acts as an anti-proliferative agent for airway smooth muscle cells [9] and human bronchial fibroblasts [14]. Currently, no information is available about the effects of formoterol in PAVSM cell proliferation as it relates to COPD-associated PH, and comparative effects of racemic formoterol vs. its (R,R) and (S,S) enantiomers on PAVSM cell proliferation are also not examined.

The mechanisms by which formoterol regulates cell proliferation are not well understood. cAMP uptake regulates Raf1-extracellular signal-regulated kinases 1/2 (ERK1/2) cascade via PKA-specific direct phosphorylation of Raf1 or PKA- and Epac1-dependent Rap1 regulation [7,15-18]. cAMP is also shown to down-regulate protein tyrosine phosphorylation in VSM cells [19]. Studies from our laboratory and others demonstrate that ERK1/2 and mammalian target of rapamycin (mTOR), downstream effectors of receptor tyrosine kinases (RTK), are two major positive regulators of PAVSM cell proliferation induced by mitogens and chronic hypoxia [20-25]. ERK1/2 is required for PDGF-, insulin- and thrombin-induced proliferation of aortic and pulmonary arterial VSM cells [22,23]; and pharmacological inhibition of MEK-ERK1/2 signaling abolishes chronic hypoxia-induced rat PAVSM cell proliferation [24]. mTOR forms two functionally distinct complexes, mTORC1 and mTORC2 [25,26]. Chronic hypoxia, PDGF, and thrombin activate mTORC1 in PAVSM and endothelial cells that, in turn, stimulates cell growth via regulation of S6 kinase 1 (S6K1) and 4 EB-P1 [20-25]. The mTORC1 inhibitor rapamycin attenuates pulmonary vascular remodeling in experimental PH [27,28] and demonstrated benefits in treatment of patients with PH [29]. mTORC2 activates serine-threonine kinase Akt via specific phosphorylation at S-473 [30]. We recently reported that chronic hypoxia and PDGF activate mTORC2 signaling that is required for proliferation of human and rat PAVSM cells [25]. The effects of formoterol and its enantiomers on ERK1/2 and mTOR signaling pathways in PAVSM cells, however, remain to be elucidated.

The goal of this study was to evaluate the effects of racemic formoterol and its (R,R) and (S,S) enantiomers on proliferation of human PAVSM cells induced by PDGF, thrombin, and chronic hypoxia, recognized triggers of PAVSM cell proliferation and vascular remodeling in COPD-associated PH [31]. We found that formoterol inhibits basal, thrombin-, and chronic hypoxia-, but not PDGF-induced proliferation of human PAVSM cells and ERK1/2 phosphorylation while having little effect on mTOR signaling. We also show that the anti-proliferative effects of formoterol require its binding with β_2_AR and that (R,R) formoterol shows improved anti-proliferative effects compared to racemic formoterol. Taken together, our data demonstrate that formoterol inhibits human PAVSM cell proliferation caused by certain PH-related stimuli and suggest that (R,R) formoterol may be considered as a potential adjuvant therapy to attenuate PAVSM cell proliferation in COPD-associated PH.

## Methods

### Cell culture

Human PAVSM cells were dissociated from pulmonary arteries from failed donor lungs that were obtained from the National Disease Research Interchange (NDRI) (Philadelphia, PA), in accordance with procedures approved by the University of Pennsylvania Committee on Studies Involving Human Beings as previously described [20,25]. Briefly, a segment of human pulmonary artery just proximal to the lung entry was removed under aseptic conditions, cleaned from connective and fat tissues and dissected as follows: the media of pulmonary artery was dissected from the adventitia and intima and subjected to an enzymatic digestion in 10 ml of buffer containing 0.2 mM CaCl_2_, 640 U/ml collagenase, 1 mg/ml soybean trypsin inhibitor and 10 U/ml elastase for approximately 60 min in a shaking water bath at 37°C. The cell suspension was filtered through 105 μm Nytex mesh, and the filtrate was washed with equal volumes of cold Ham's F-12 medium (Life Technologies, Grand Island, NY) supplemented with 10% FBS (HyClone, Logan, UT). Cells were plated on tissue culture plates covered with Vitrogen (Cohesion Technologies Inc., Palo Alto, CA). Cells were cultured in Ham's F-12 media supplemented with 10% FBS (Becton Dickinson, Bedford, MA), 100 U/ml penicillin, and 0.1 mg/ml streptomycin.

For chronic hypoxia experiments, cells were maintained either in normoxic (21% O_2_, 5% CO_2_) or in hypoxic (1% O_2_, 5% CO_2_) conditions for 7 days in complete media and then for 48 h in serum-free media supplemented with 0.1% BSA. 1% O_2_ was used as we described [25] to reproduce the tissue oxygen levels of moderate chronic hypoxia [32,33] based on data obtained from the rat chronic hypoxia model of PH [34,35]. To avoid re-oxygenation, fresh complete or serum-free media was pre-equilibrated at 1% O_2_, before adding to PAVSM cells grown under hypoxia. Primary human PAVSM cells in subculture during the second through tenth cell passages were used. All experiments were performed using a minimum of three different cell cultures. Each human PAVSM cell culture was established using pulmonary arterial tissue from a single human donor.

### DNA synthesis analysis

Cells grown under normoxia or chronic hypoxia were serum deprived for 48 h, treated with 0.1, 1, or 10 ng/ml PDGF-BB, 1 U/ml thrombin, 0.2, 2, or 20 μM racemic, (R,R), or (S,S) formoterol, or diluent for 18 h followed by DNA synthesis analysis using the BrdU incorporation assays as we described [25,36-38]. Briefly, cells were incubated with 10 μM BrdU for 24 h, fixed with 3.7% paraformaldehyde (Polysciences, Inc., Warrington, PA) for 15 min and permeabilized with 0.1% Triton X-100 for 30 min at room temperature. Following denaturation of DNA with 4 N HCl (3 min at room temperature), incubated for 1 h at 37^o^C with 2 μg/ml murine anti-BrdU primary (Becton Dickinson, San Jose, CA) and 10 μg/ml Texas Red-conjugated anti-mouse secondary (Jackson ImmunoResearch Laboratories, West Grove, PA) antibodies for 1 h at 37°C to detect BrdU-positive cells. To detect the total number of nuclei, cells were incubated with 1 μg/ml DAPI. Then, cells were visualized using Eclipse Nikon TE2000E fluorescent microscope (200x magnification), and automatic counts of BrdU-positive and total number of cells were performed using Image-Pro Plus 5.1 software.

### Immunoblot analysis

Whole cell lysates were prepared using a buffer comprised of 40 mM HEPES (pH 7.5), 120 mM NaCl, 1 mM EDTA, 10 mM sodium pyrophosphate (Na_4_P_2_O_7_ x 10H_2_O), 10 mM β-glycerophosphate, 50 mM NaF, 1.5 mM Na_3_VO_4_, 1% Triton X-100, EDTA-free inhibitor cocktail. Protein contents were measured using a Bio-Rad protein assay reagent kit. Equal amounts of lysate, adjusted for protein content, were subjected to SDS-PAGE and then immunoblot analysis. The blots were exposed to anti-phospho-S6 ribosomal protein, anti-total S6, anti-phospho Akt Ser-473, anti-total Akt, anti-phospho ERK, anti-total ERK, anti-phospho S6K1 T-389, and anti-total S6K1 antibodies (Cell Signaling Technology, Inc., Beverly, MA). All antibodies were in 20 mM Tris (pH 7.5), 150 mM NaCl (TBS) plus 0.5% Tween 20 (TBST), and all incubations were for overnight at 4°C. After 3 washes in TBST, the nitrocellulose membranes were exposed to horseradish peroxidase-conjugated secondary antibody (Boehringer-Mannheim, Indianapolis, IN), washed five times in TBST and visualized using enhanced chemiluminescence (ECL) (Amersham, Arlington Heights, IL).

### Data analysis

Data points from each condition are represented as the mean values ± SE. Three independent repetitions were performed for each experimental condition. Statistical analysis was performed using StatView software. Statistically significant differences among groups were assessed with the analysis of variance (ANOVA) (Bonferroni-Dunn) with values of p < 0.05 sufficient to reject the null hypothesis for all analyses. All experiments were designed with matched control conditions within each experiment to enable statistical comparison as paired samples.

## Results

### Formoterol inhibits basal and thrombin-, but not PDGF-induced human PAVSM cell proliferation

**Figure 1:**
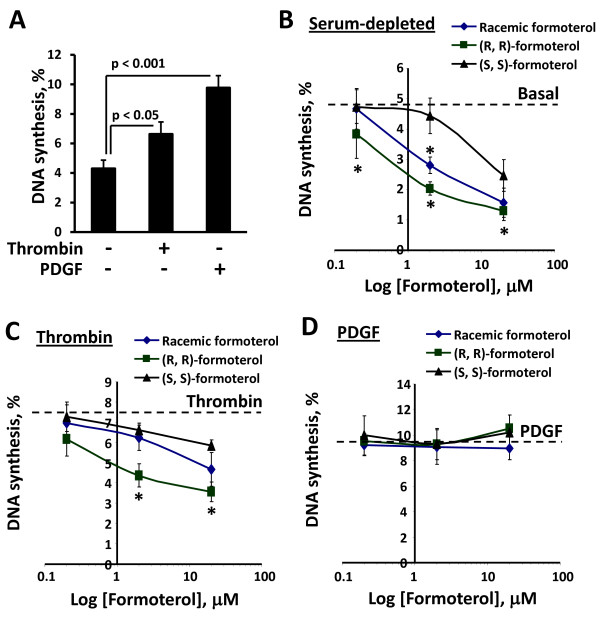
**Formoterol inhibits basal and thrombin-, but not PDGF-induced DNA synthesis in human PAVSM cells. A: C**ells serum-deprived for 48 h were treated for 18 h with 1 U/ml thrombin, 10 ng/ml PDGF, or diluent followed by DNA synthesis analysis using the BrdU incorporation assay. Data represent a percentage of BrdU-positive cells per total number of cells taken as 100%. Data are mean values ± SE from three independent experiments. A minimum of 200 cells per each condition were analyzed in each experiment. **B-D:** Cells serum-deprived for 48 h were treated with 0.2, 2, 20 μM (R,R)-, (S,S)-, racemic formoterol, or diluent in the presence of vehicle (**B**), 1 U/ml thrombin (**C**) or 10 ng/ml PDGF (**D**) followed by DNA synthesis analysis using the BrdU incorporation assay. Data are means ± SE from three separate experiments, n = 3 for each experimental condition. *p < 0.001 for formoterol vs. diluent and for thrombin + (R,R) formoterol vs. thrombin by ANOVA (Bonferroni-Dunn). A minimum of 200 cells per condition were analyzed in each experiment.

Because formoterol inhibits proliferation of human bronchus fibroblasts and airway smooth muscle cells [9,14], we examined the effects of formoterol and its enantiomers on the proliferation of human PAVSM cells under serum-depleted conditions and in the presence of thrombin and PDGF, confirmed PAVSM mitogens that are involved in PH pathogenesis [20,39,40]. Consistent with our published data [20], serum-deprived PAVSM cells had modest levels of DNA synthesis that were significantly increased by treatment with PDGF and thrombin (Figure 1A). Racemic formoterol inhibited DNA synthesis in serum-deprived human PAVSM cells in a concentration-dependent manner (Figure 1B). (R,R) formoterol had a greater inhibitory effect on human PAVSM cell proliferation compared to racemic formoterol (IC_50_ ~ 1 μM and ~ 4 μM, respectively). (S,S)-formoterol had modest inhibitory effect (IC_50_ ~ 20 μM) and attenuated DNA synthesis only when used in maximal dose. (R,R) and, to a lesser extent, racemic formoterol significantly inhibited thrombin-induced human PAVSM cell proliferation (IC_50_ for (R,R) formoterol ~ 20 μM), and (S,S) formoterol had no significant effect (Figure 1C). Both racemic and (S,S) formoterol did not provide 50% inhibition of cell proliferation even when administrated in high doses (20 μM). Interestingly, racemic formoterol and both formoterol enantiomers had no effect on PDGF-induced proliferation of human PAVSM cells (Figure 1D). These data demonstrate that formoterol inhibits basal and thrombin-induced, but not PDGF-stimulated human PAVSM cell proliferation and suggest that growth inhibitory effects of formoterol depend predominantly on its (R,R), but not (S,S) enantiomer.

### Formoterol inhibits PAVSM cell proliferation caused by chronic hypoxia exposure

**Figure 2:**
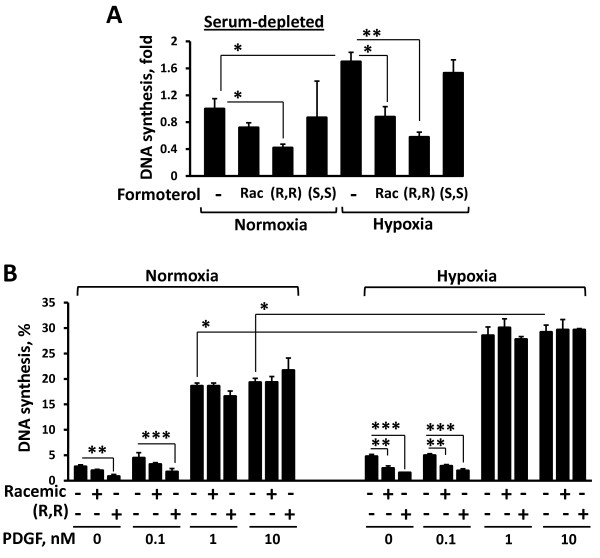
**Formoterol inhibits chronic hypoxia-induced human PAVSM cell proliferation.** Cells grown under chronic hypoxia (1% O_2_) or normoxia (21% O_2_) for 7 days were serum-deprived for 48 h, treated with 10 μM (R,R)-, (S,S)-, racemic formoterol, or diluent (A), or 0.1, 1, 10 ng/ml PDGF, or diluent in the absence or presence of 10 μM (R,R) and racemic formoterol (B), and then DNA synthesis analysis using the BrdU incorporation assay was performed. DNA synthesis was measured as a percentage of the BrdU-positive cells per total number of cells. Data are means ± SE by ANOVA (Bonferroni-Dunn) from three independent experiments; n = 3 for each experimental condition. A minimum of 200 cells per condition were analyzed in each experiment. **A:** *p < 0.01 for diluent-treated cells under normoxia vs. hypoxia and for racemic formoterol vs. (R,R) formoterol under normoxia; **p < 0.001 for racemic formoterol vs. (R,R) formoterol under hypoxia. **B:** *p < 0.001 for hypoxia vs. normoxia; **p < 0.01 for diluent vs. (R,R) formoterol under normoxia; for diluent vs. racemic formoterol under hypoxia; and for 0.1 ng/ml PDGF vs. 0.1 ng/ml PDGF + racemic formoterol under hypoxia; ***p < 0.001 for 0.1 ng/ml PDGF vs. 0.1 ng/ml PDGF + (R,R) formoterol under normoxia; for diluent vs. (R,R) formoterol under hypoxia; and for 0.1 ng/ml PDGF + (R,R) formoterol under hypoxia.

Because chronic hypoxia is one of the confirmed triggers of PAVSM cell proliferation and pulmonary vascular remodeling in PH [2,41], we next examined whether formoterol modulates chronic hypoxia-induced PAVSM cell proliferation. As we demonstrated previously [25], chronic hypoxia increased basal and PDGF-induced human PAVSM cell proliferation compared to normoxia-maintained cells (Figure 2A and B, respectively). Importantly, (R,R)-formoterol inhibited chronic hypoxia-induced PAVSM cell proliferation by the level comparable to normoxia-maintained cells, racemic formoterol had lesser inhibitory effect, and no significant differences in cell proliferation were observed in (S,S) formoterol- vs. diluent-treated PAVSMC under chronic hypoxia (Figure 2A). Interestingly, 10 μM (R,R) and racemic formoterol attenuated PAVSM cell proliferation in the presence of 0.1 ng/ml PDGF, but had little effect on the proliferation induced by higher PDGF doses (1 and 10 ng/ml) either under chronic hypoxia or normoxia (Figure 2B) demonstrating that PDGF-induced PAVSM cell proliferation is not susceptible to formoterol. Taken together, these data demonstrate that formoterol attenuates basal, but not PDGF-induced human PAVSM proliferation under chronic hypoxia and that formoterol acts predominantly through its (R,R) enantiomer.

### Binding with β_2_AR is required for formoterol-induced inhibition of human PAVSM cell proliferation

**Figure 3:**
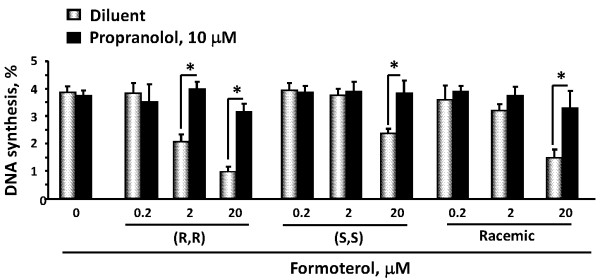
**β_2_AR blocker propranolol abolishes formoterol-induced inhibition of DNA synthesis in human PAVSM cells.** Cells were serum-deprived for 48 h, treated with 0.2, 2, 20 mM (R,R), (S,S), racemic formoterol, or diluent for 18 h in the presence (black bars) or absence (grey bars) of 10 μM propranolol followed by DNA synthesis analysis using the BrdU incorporation assay. Data represent mean values ± SE from two independent experiments, three repetitions per each experiment. *p < 0.001 for formoterol vs. formoterol + propranolol by ANOVA (Bonferroni-Dunn).

Since the bronchodilatory functions of formoterol require binding of its (R,R) enantiomer with specific β_2_AR [42], we next examined whether binding with β_2_AR contributes to anti-proliferative effects of formoterol in human PAVSM cells. Serum-deprived cells were treated with the β_2_AR blocker propranolol in the presence of 0.2-20 μM of (R,R), (S,S) and racemic formoterol, or diluent and then subjected to DNA synthesis analysis using the BrdU incorporation assay. As seen in Figure 3 (grey bars), (R,R), racemic, and, to a lesser extent, (S,S)-formoterol inhibited DNA synthesis in human PAVSM cells depending on agent concentration. Importantly, propranolol completely reversed inhibitory effects of racemic, (R,R)-, and (S,S)-formoterol on PAVSM cell proliferation (Figure 3, black bars). These data demonstrate that binding with β_2_AR is required for formoterol-dependent inhibition of human PAVSM cell proliferation.

### (R,R) formoterol inhibits basal and thrombin-, but not PDGF-induced ERK1/2 phosphorylation in human PAVSM cells

**Figure 4:**
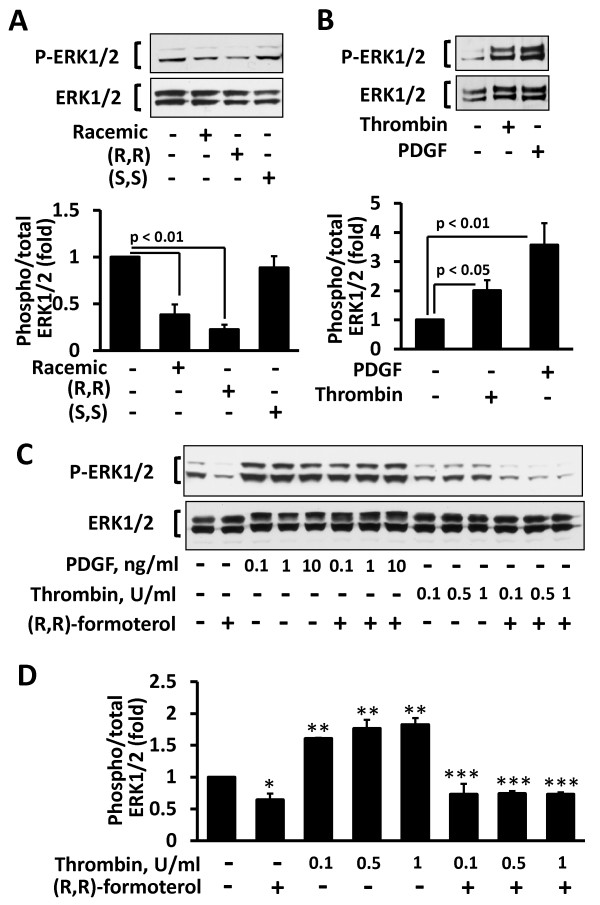
**Differential effects of formoterol on basal, thrombin- and PDGF-induced ERK1/2 phosphorylation in human PAVSM cells. A:** (R,R) and racemic, but not (S,S) formoterol inhibit ERK1/2 phosphorylation in human PAVSM cells. Cells serum-deprived for 48 h were treated with 10 μM (R,R), (S,S), racemic formoterol or diluent for 30 min followed by immunoblot analysis with anti-phospho ERK1/2 and anti-total ERK1/2 antibodies. **Top panel:** Images are representative of three independent experiments. **Bottom panel:** Statistical analysis of three separate experiments, n = 3 for each experimental condition. Data represent mean values ± SE by ANOVA (Bonferroni-Dunn). Phospho-ERK/total ERK ratio for diluent-treated cells was taken as one fold. **B:** Cells serum-deprived for 48 h were treated with 1 U/ml thrombin, 10 ng/ml PDGF, or diluent for 30 min followed by immunoblot analysis with anti-phospho ERK1/2 and anti-total ERK1/2 antibodies. **Top panel:** Representative images from three independent experiments. **Bottom panel:** Statistical analysis of three independent experiments; n = 3 for each experimental condition. Data are mean values ± SE by ANOVA (Bonferroni-Dunn). Phospho-ERK/total ERK ratio for diluent-treated cells was taken as one fold. **C, D: **(R,R) formoterol inhibits thrombin-, but not PDGF-induced ERK1/2 phosphorylation in human PAVSM cells. Cells were serum-deprived for 48 h, incubated for 30 min with 10 μM (R,R), (S,S), or racemic formoterol in the presence or absence of 10 ng/ml PDGF, and then immunoblot analysis with anti-phospho-ERK1/2 and anti-total ERK1/2 antibodies was performed. **C:** Representative images. **D:** Statistical analysis of three separate experiments. Data are mean ± SE by ANOVA (Bonferroni-Dunn). Phospho/total ERK ratio for diluent-treated cells was taken as one fold. *p < 0.05 for diluent vs. (R,R) formoterol; **p < 0.01 for diluent vs. thrombin; ***p < 0.01 for thrombin vs. thrombin + (R,R) formoterol.

Because ERK1/2 signaling is involved in human PAVSM cell proliferation, we next determined the effects of formoterol and its enantiomers on ERK1/2 activatory phosphorylation. As seen in Figure 4A, (R,R) and, to a lesser extent, racemic, but not (S,S) formoterol inhibited basal ERK1/2 phosphorylation. Because PDGF and thrombin, but not chronic hypoxia, markedly activate ERK1/2 in PAVSM cells [20,25,43,44] (Figure 4B), ERK1/2 activation and since (R,R) formoterol demonstrated maximal inhibitory effects on human PAVSM cell proliferation and basal ERK1/2 phosphorylation compared to racemic and (S,S) formoterol, we next evaluated effects of (R,R) formoterol on ERK1/2 phosphorylation induced by PDGF and thrombin. As seen in Figure 4C, D, (R,R) formoterol abrogated thrombin-induced ERK1/2 phosphorylation while having little effect on ERK1/2 phosphorylation induced by PDGF over a range of concentrations (0.1 - 10 ng/ml). Similarly, both (R,R) and racemic formoterol failed to inhibit PDGF-induced ERK1/2 phosphorylation either under normoxia or chronic hypoxia conditions (data not shown). Taken together, these data demonstrate that racemic and (R,R) formoterol down-regulate basal and thrombin-induced, but not PDGF-dependent ERK1/2 phosphorylation in human PAVSM cells.

### Formoterol has no effect on mTOR signaling in human PAVSM cells

**Figure 5:**
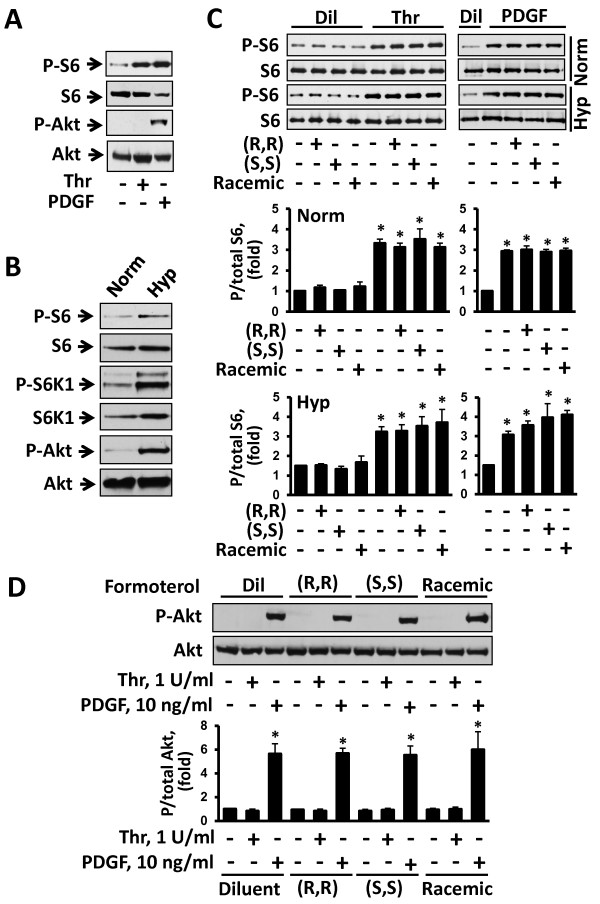
**Formoterol does not affect mTORC1 and mTORC2 activation by chronic hypoxia, thrombin and PDGF. A:** Human PAVSM cells serum-deprived for 48 h were treated with 1 U/ml thrombin (thr), 10 ng/ml PDGF, or diluent (dil) for 30 min, and then immunoblot analysis with anti-phospho S6, anti-total S6, anti-phospho S473-Akt, and anti-total Akt was performed. Images are representative of three independent experiments. **B:** Chronic hypoxia promotes S6, S6K1, and S473-Akt phosphorylation in human PAVSM cells. Human PAVSM cells serum-deprived for 48 h maintained under chronic hypoxia (hyp) or normoxia (norm) were subjected to immunoblot analysis with specific antibodies to detect indicated proteins. Images are representative of three independent experiments. **C, D:** Formoterol has no effect on S6 and S473-Akt phosphorylation. C: Serum-deprived cells maintained under hypoxia or normoxia were incubated for 30 min with 10 μM (R,R)-, (S,S), or racemic formoterol, treated with different concentrations of thrombin and PDGF, or diluent, and then immunoblot analysis with anti-phospho-S6 and anti-total S6 antibodies was performed. Representative images (top) and statistical analysis (bottom) of three independent experiments. Data represent P/total S6 ratio (n = 3 for each experimental condition). P/total S6 ratio for diluent-treated normoxia-exposed cells was taken as one fold). Data are means ± SE by ANOVA (Bonferroni-Dunn). *p < 0.001 for thrombin vs. diluent, thrombin + (R,R), (S,S) and racemic formoterol vs. (R,R), (S,S) and racemic formoterol, respectively. **D:** Immunoblot analysis of serum-deprived normoxia-maintained cells treated as described above was performed to detect P-S473-Akt and total Akt levels. Representative images (top) and data analysis (bottom) from three separate experiments. Data represent P-S473/total Akt ratio (n = 3 for each experimental condition) normalized by P/total Akt for diluent-treated cells (taken as one fold). Data are means ± SE by ANOVA (Bonferroni-Dunn). *p < 0.001 for PDGF vs. diluent, PDGF + (R,R) formoterol vs. (R,R) formoterol, PDGF + (S,S) formoterol vs. (S,S) formoterol, and PDGF + racemic formoterol vs. racemic formoterol.

Next, we examined whether formoterol modulates mTOR signaling in human PAVSM cells by assessing mTORC1-specific ribosomal protein S6 and mTORC2-specific S473-Akt phosphorylation. Consistent with our published data [20,25], serum-deprived human PAVSM cells had modest S6 phosphorylation levels that were markedly increased by thrombin and PDGF (Figure 5A). In contrast, PDGF, but not thrombin, promoted S473-Akt phosphorylation (Figure 5A) suggesting that both PDGF and thrombin activate mTORC1 signaling in human PAVSM cells while only PDGF increases mTORC2 activity. As we reported previously [25], exposure to chronic hypoxia led to a marked increase in mTORC1-dependent P-S6K1 and P-S6 and mTORC2-dependent P-S473-Akt (Figure 5B) demonstrating that chronic hypoxia activates both mTORC1 and mTORC2 pathways in human PAVSM cells. Interestingly, racemic formoterol and both (R,R) and (S,S) formoterol enantiomers had no effect on basal, PDGF- and thrombin-induced S6 phosphorylation either under normoxia or chronic hypoxia (Figure 5C). PDGF-induced S473-Akt phosphorylation was also comparable in diluent-, (R,R)-, (S,S)-, and racemic formoterol-treated cells (Figure 5D). Collectively, these data demonstrate that formoterol has little effect on mTORC1 and mTORC2 signaling pathways in human PAVSM cells.

## Discussion

PH is a progressive disease with poor prognosis, the pathological manifestations of which include vasoconstriction and pulmonary vascular remodeling [5,45]. PH may be familial, idiopathic, or associated with other diseases. Notably, patients with COPD-associated PH have higher morbidity and reduced survival compared to other COPD patients [3,4]. Pulmonary vascular remodeling in PH is associated with marked medial thickening of small muscular PAs due, at least in part, to increased proliferation of PAVSM cells. PAVSM cell proliferation in COPD-associated PH is caused by multiple factors including persistent hypoxia and increased production of growth factors and pro-inflammatory cytokines [2,31,46]. Our study demonstrates that β_2_AR agonist formoterol inhibits proliferation of PAVSM cells induced by thrombin and chronic hypoxia, but not PDGF. Anti-proliferative activity of formoterol requires binding with β_2_AR, and (R,R) enantiomer of formoterol shows improved anti-growth effects compared to racemic and (S,S) formoterol. We also report that formoterol inhibits basal and thrombin-induced activation of ERK1/2, but has no effect on mTOR signaling in human PAVSM cells.

Deregulated PAVSM cell proliferation is one of the major pathological components of pulmonary vascular remodeling in PH. The long-term β_2_AR agonist formoterol, which is currently in use as bronchodilator in COPD, shows anti-proliferative activities in human airway smooth muscle cells and bronchus fibroblasts [9,14]. In the present study, we explored effects of formoterol on human PAVSM cell proliferation caused by different stimuli involved in PH pathogenesis [2,31,47] and found that the growth-inhibitory potency of formoterol highly depends on extracellular stimuli. Thus, formoterol and, especially, its (R,R) enantiomer, inhibited proliferation of non-stimulated PAVSM cells under chronic hypoxia, decreased thrombin-induced proliferation, but had no significant effect on PDGF-dependent PAVSM cell growth.

The mechanisms of β_2_AR-dependent regulation of cell proliferation are relatively unexplored and appear to be highly cell type-specific. β_2_AR-induced PKA activation, while inhibiting proliferation in the majority of cell types including vascular smooth muscle cells [8-11,48,49] stimulates proliferation of human uveal melanoma cells [50] and cardiomyocyte hypertrophy [51]. Activation of β_2_AR-cAMP signaling also up-regulates Epac1, the predominant Epac isoform in VSM cells [7]. Epac1 synergizes with PKA in inhibiting VSM cell proliferation [7], but increases DNA synthesis in macrophages and prostate cancer cells [52,53]. Our data show that formoterol-dependent inhibition of human PAVSM cells requires its binding with β_2_AR. Accordingly, (R,R) formoterol, which has much higher receptor affinity and greater potency to induce β_2_AR-G_s_-dependent cAMP production and PKA activation compared to (S,S) formoterol [8,54], demonstrates greater anti-proliferative effects than formoterol racemate while (S,S) formoterol has modest effects on PAVSM cell proliferation.

Emerging evidence shows that, in addition to classical G_s_-cAMP pathway, β_2_AR may interact with G_i_ proteins that, in contrast to G_s_, leads to reduction of cAMP levels and inhibition of PKA-dependent signaling [55]. Currently, no evidence exists about involvement of G_i_ in PAVSM cell proliferation and pulmonary vascular remodeling in PH. In contrast, G_s_-dependent activation of cAMP-PKA signaling is well documented in human ASM and PAVSM cells upon formoterol treatment and is required for bronchodilatory and vasodilatory effects of formoterol on COPD patients and for inhibition of SM cell proliferation [7-11,56]. G_i_ overexpression, however, has been reported in heart and aorta of spontaneously hypertensive rats; and G_i_ suppression with pertussis toxin attenuated development of high blood pressure in this model [55,57-59] suggesting differential mechanisms of β_2_AR signaling in heart vs. pulmonary vasculature.

The signaling pathways underlying formoterol-dependent inhibition of cell proliferation are not well evaluated. We and others previously demonstrated that thrombin promotes human PAVSM cell proliferation via ERK1/2 signaling while PDGF acts via activation of two major pro-proliferative pathways, PI3K-mTOR and MEK-ERK1/2 [60]. In the majority of cells, including VSM, cAMP-dependent activation of PKA and Epac inhibits ERK1/2 signaling via modulating activities of small GTPases Raf-1 and Rap-1 downstream of Ras [15,61,62] clearly demonstrating functional cross-talk between ERK1/2 and β_2_AR-cAMP cascades.

Interestingly, we found that formoterol markedly inhibits thrombin-, but not PDGF-induced ERK1/2 phosphorylation. A possible explanation is that PDGF, in addition to ERK1/2, also promotes strong up-regulation of PI3K signaling [20,25], and we found that PI3K is insensitive to formoterol. PI3K stimulates ERK1/2 activation via Raf-1 and Rap-1 in a Ras-independent manner [63,64] and can counter-balance formoterol-β_2_AR-cAMP-dependent ERK1/2 inhibition. Indeed, our data show that formoterol markedly inhibits ERK1/2 phosphorylation in chronic hypoxia-exposed PAVSM cells, in which mTOR activation and proliferation occur in a PI3K-independent manner [25].

Chronic hypoxia-induced proliferation of PAVSM cells requires expression of hypoxia-inducible factor 1 α (HIF1α), which plays a critical role in PAVSM remodeling in human and experimental PH [31,65]. Notably, ERK1/2 up-regulates HIF1α transcriptional activity via direct phosphorylation that promotes HIF1α nuclear translocation or via regulating binding of HIF1α with its major co-activator p300/cAMP response element-binding protein (CBP) [66-69]. Thus, formoterol may inhibit chronic hypoxia-induced PAVSM cell proliferation via down-regulation of ERK1/2-dependent HIF1α transcriptional activity.

Although much less is known about the regulation of the mTOR signaling pathway by cAMP/PKA, it is shown that cAMP elevation inhibits mTORC1/S6K1 in T lymphocytes [70], but not in CCL39 fibroblasts [71] suggesting that effects of cAMP/PKA on mTOR activation are cell type-specific. Our data show that formoterol has no effect on activation of mTORC1 and mTORC2 signaling pathways caused by either PDGF or chronic hypoxia. These data indicate that mitogen- and chronic hypoxia-induced mTOR activation in human PAVSM cells is β_2_AR-independent.

Taken together, our study demonstrates that formoterol inhibits basal, chronic hypoxia- and thrombin-, but not PDGF-induced human PAVSM cell proliferation potentially via β_2_AR-dependent inhibition of ERK1/2 signaling pathway; and that anti-proliferative activity of formoterol is provided predominantly by its (R,R) enantiomer. This data suggests that (R,R) formoterol, while having a limited effect as a single agent, may be considered as a potential adjuvant therapy for COPD-associated PH.

## Conclusions

Collectively, our data demonstrate that the β_2_AR agonist formoterol inhibits basal, thrombin-, and chronic hypoxia-induced ERK1/2 activation and proliferation of human PAVSM cells, but has little effect on PDGF-induced ERK1/2 activation, proliferation and mTOR signaling. We also show that formoterol acts predominantly through its (R,R) enantiomer and that anti-proliferative effects of formoterol require β_2_AR binding. These data suggest that (R,R) formoterol inhibits human PAVSM cell proliferation in certain conditions and may be considered as a potential adjuvant therapy for COPD-associated PH.

## Abbreviations

ANOVA: Analysis of variance; β_2_AR: β_2_-adrenergic receptor; BrdU: 5-bromo-2'-deoxyuridine; COPD: Chronic obstructive pulmonary disease; DAPI: 4',6-diamidino-2-phenylindole; ERK1/2: Extracellular signal-regulated kinases ½; HIF1α: hypoxia-inducible factor 1 α; mTOR: Mammalian target of rapamycin; MAPK: Mitogen-activated protein kinase; MEK: MAP kinase kinase; NDRI: National Disease Research Interchange; PAs: pulmonary arteries; PAVSM: Pulmonary arterial vascular smooth muscle; PDGF: platelet-derived growth factor; PH: Pulmonary hypertension; PKA: Protein kinase A; RTK: Receptor tyrosine kinase; S6K1: p70 S6 kinase 1.

## Competing interests

The authors declare that they have no competing interests.

## Authors’ contributions

EAG performed statistical analysis, participated in study coordination and drafted the manuscript. ISK carried out the immunoblots and participated in the statistical analysis of immunoblots. DAG carried out DNA synthesis experiments and participated in the statistical analysis of DNA synthesis data. VPK designed and coordinated the study and revised the manuscript. All authors read and approved the final manuscript.
